# Neighborhood Characteristics to Enhance Social Connectedness for Older People With Dementia

**DOI:** 10.1093/geroni/igaf122.2120

**Published:** 2025-12-31

**Authors:** Xiaoyi Zeng, Yuanjin Zhou, Lailea Noel, Catherine Cubbin, Namkee Choi

**Affiliations:** The University of Texas at Austin, Austin, Texas, United States; The University of Texas at Austin, Austin, Texas, United States; The University of Texas at Austin, Austin, Texas, United States; The University of Texas at Austin, Austin, Texas, United States; The University of Texas at Austin, Austin, Texas, United States

## Abstract

This qualitative study explored the perspectives of older people with mild cognitive impairment (MCI) or dementia and their care partners on: (1) neighborhood-level facilitators and barriers influencing social connectedness, and (2) essential characteristics of dementia-friendly neighborhoods addressing the social connectedness needs of older people with dementia. Seventeen semi-structured interviews, each lasting an average of 77 minutes, were conducted with nine older adults with MCI or mild dementia (mean age = 76.4 years, 55.6% female) and eight care partners (mean age = 68.5 years, 75% female) in a large metropolitan area in a Southwestern state in June to August, 2024. Thematic analysis steps were employed for data analysis. Five key facilitators that emerged were: (1) safe and walkable environments, (2) access to essential services and social activities, (3) strong neighborhood ties, (4) stable and familiar surroundings, and (5) dementia-friendly community programs. Barriers were: (1) safety concerns, (2) transportation, (3) limited access to suitable activities, (4) lack of meaningful interactions with others in the neighborhood, and (5) public stigma surrounding dementia and aging. Participants emphasized neighborhood environments that protect older people with dementia’s autonomy, interests, safety, and accessibility. Findings underscore the importance of expanding inclusive and affordable community programs for older people with dementia and empowering care partners and neighbors to facilitate social connectivity through senior-friendly transportation, assistance with adaptations to changes in the neighborhood environment, and public stigma reduction education. These community-driven insights provide actionable guidance for developing and advancing dementia-friendly neighborhoods that foster social connectedness and support aging in place.

